# Investigation of Different Library Preparation and Tissue of Origin Deconvolution Methods for Urine and Plasma cfDNA Methylome Analysis

**DOI:** 10.3390/diagnostics13152505

**Published:** 2023-07-27

**Authors:** Nicholas Kueng, Daniel Sidler, Vanessa Banz, Carlo R. Largiadèr, Charlotte K. Y. Ng, Ursula Amstutz

**Affiliations:** 1 Department of Clinical Chemistry, Inselspital, Bern University Hospital, University of Bern, 3010 Bern, Switzerland; 2 Graduate School for Cellular and Biomedical Sciences, University of Bern, 3012 Bern, Switzerland; 3 Department of Nephrology and Hypertension, Inselspital, Bern University Hospital, University of Bern, 3010 Bern, Switzerland; 4 Department of Visceral Surgery and Medicine, Inselspital, Bern University Hospital, University of Bern, 3010 Bern, Switzerland; 5 Department for BioMedical Research (DBMR), University of Bern, 3008 Bern, Switzerland

**Keywords:** cfDNA, methylation sequencing, tissue of origin, enzymatic conversion, transplant, urine, plasma, single-stranded library preparation

## Abstract

Methylation sequencing is a promising approach to infer the tissue of origin of cell-free DNA (cfDNA). In this study, a single- and a double-stranded library preparation approach were evaluated with respect to their technical biases when applied on cfDNA from plasma and urine. Additionally, tissue of origin (TOO) proportions were evaluated using two deconvolution methods. Sequencing cfDNA from urine using the double-stranded method resulted in a substantial within-read methylation bias and a lower global methylation (56.0% vs. 75.8%, *p* ≤ 0.0001) compared to plasma cfDNA, both of which were not observed with the single-stranded approach. Individual CpG site-based TOO deconvolution resulted in a significantly increased proportion of undetermined TOO with the double-stranded method (urine: 32.3% vs. 1.9%; plasma: 5.9% vs. 0.04%; *p* ≤ 0.0001), but no major differences in proportions of individual cell types. In contrast, fragment-level deconvolution led to multiple cell types, with significantly different TOO proportions between the two methods. This study thus outlines potential limitations of double-stranded library preparation for methylation analysis of cfDNA especially for urinary cfDNA. While the double-stranded method allows jagged end analysis in addition to TOO analysis, it leads to significant methylation bias in urinary cfDNA, which single-stranded methods can overcome.

## 1. Introduction

Cell-free DNA (cfDNA) is released into body fluids from cells of various tissues through different processes [1]. The cell-type specific methylation signature mostly occurring at CpG sites is preserved in cfDNA, and thus can be utilized to gain information about its tissue of origin (TOO) [2]. Various studies using TOO deconvolution of cfDNA based on tissue-specific methylation patterns have shown that the composition of cfDNA is dynamic and can be influenced by physical activity [3] or diseases [4,5,6]. Current clinical implementations of cfDNA as a biomarker use tissue-specific genome sequence differences to infer the tissue of origin, which limits its use to applications in the context of cancer [7], allograft monitoring [8] and non-invasive prenatal testing [9]. Methylation-based TOO deconvolution expands the potential clinical utility of cfDNA beyond tissues with genotype differences to pathologies without any DNA sequence alterations, like neurogenerative disorders or chronic inflammatory diseases, such as rheumatoid arthritis.

**Figure 1:**
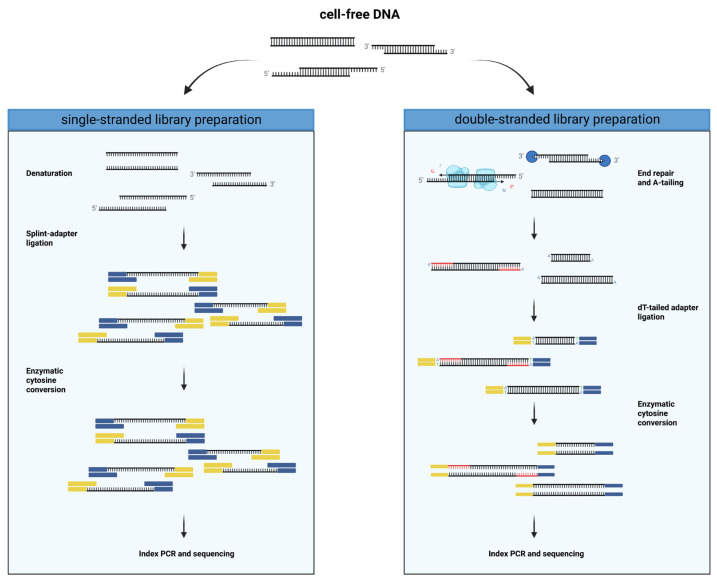
Overview of the library preparation workflows compared in this study.

Sodium bisulfite-mediated cytosine conversion has long been the gold standard for methylation analysis, despite its drawbacks of DNA fragmentation [10], DNA loss and unbalanced nucleotide composition [11]. To overcome these limitations, a new method using enzymatic-based cytosine conversion has been developed [12]. This method utilizes ten-eleven translocation (TET) methylcytosine dioxygenases-mediated oxidation of 5-methylcytosine (5-mC) and 5-hydroxymethylcytosine (5-hmC) to protect them from subsequent deamination of unmethylated cytosines by APOBEC3A (apolipoprotein B mRNA editing enzyme catalytic subunit 3A). This approach enables the detection of 5-mC and 5-hmC with minimized DNA damage and loss, which is of particular relevance for applications with limited starting material, such as cfDNA. It allows for performing the conversion after adapter ligation to preserve the fragmentation pattern. Enzymatic conversion-based methylation sequencing has been successfully applied to cfDNA methylome analysis [12,13]. So far, this enzymatic conversion method has only been used in conjunction with a double-stranded library preparation approach. This is the most common type of library preparation method that involves the creation of blunt ends through the incorporation of bases at 5′ overhangs and digestion of 3′ overhangs (Figure 1). This end repair step is followed by an A-tailing reaction, which is required for the ligation of sequencer-specific dT-tailed adapters to the DNA fragment. One of the more recently developed alternative library preparation methods called SRSLY [14], which is commercially available, uses an adapter based on a modified splint-adapter design proposed by Gansauge et al. [15]. This technique allows the ligation of adapters to single-stranded DNA fragments and has been shown to better capture the diversity of DNA species in cfDNA, such as nicked, jagged and short single-stranded subnucleosomal cfDNA [16]. Moreover, it preserves cfDNA end motives, as it does not require blunt ends for adapter ligation commonly obtained through end repair. Importantly, this end repair includes the incorporation of unmethylated cytosines, creating a bias by altering the original methylation patterns. While both single-stranded and double-stranded library preparation methods have been used for cfDNA methylome analysis with TOO deconvolution, literature reporting on the robustness and possible pitfalls of these methods with cfDNA from different body fluids and the choice of deconvolution algorithms is limited.

This pilot study aimed to investigate technical differences and potential biases of single-stranded and double-stranded library preparation in conjunction with enzymatic-based conversion for whole genome methylation sequencing and their impact on TOO deconvolution of cfDNA from both plasma and urine.

## 2. Materials and Methods

### 2.1. Patient Recruitment and Sample Collection

We recruited liver and kidney transplant recipients at the Inselspital (Bern University Hospital, Bern, Switzerland) between August 2019 and August 2020 after approval from the ethics committee of the Canton of Bern, Switzerland (2019-00730). Whole blood and urine samples were collected once at a routine follow-up appointment or at up to three time points within the first week after transplantation. Moreover, plasma samples from two anonymized healthy volunteers without a history of organ transplantation or cancer and no recent (<3 months) blood transfusion or pregnancy were collected.

Venous blood and urine were collected and processed as previously described [17].

### 2.2. cfDNA Extraction

After thawing the plasma at 4 °C, cfDNA was isolated with the QIAamp Circulating Nucleic Acid Kit (Qiagen, Hilden, Germany), according to the protocol provided by the manufacturer, as previously described [17].

Urinary cfDNA was extracted from 15–50 mL of urine after thawing at room temperature (RT) either with the Quick-DNA Urine Kit (Zymo Research, Irvine, CA, USA) as previously described [17] or using a quaternary ammonium anion exchange resin-based Q Sepharose method based on the method published by Dudley et al. [18].

For the Q Sepharose method, 300 µL of Q Sepharose^®^ Fast Flow slurry (Cytiva, Marlborough, MA, USA) was added for every 10 mL of urine and incubated on a rolling drum at RT for 30 min. The resins were pelleted at 1800× *g* for 5 min at RT, and the supernatant was discarded. The pellet was resuspended in 1 mL 0.3 M LiCl/10 mM NaOAc (pH 5.5), transferred to a Micro Bio-Spin Chromatography column (Bio-Rad Laboratory, Hercules, CA, USA) and centrifuged at 800× *g* for 1 min. The resins were washed three times with 670 µL of 0.3 M LiCl/10 mM NaOAc (pH 5.5), while centrifuging at 800× *g* for 1 min between each wash step. The cfDNA was eluted twice with 670 µL 2 M LiCl/10 mM NaOAc (pH 5.5) (800× *g*, 1 min). Afterwards, 4 mL of 85% ethanol was added to the eluate, and the mixture was incrementally (700 uL) transferred to a QIAquick spin column (Qiagen, Hilden, Germany), with centrifugation steps in between (800× *g*, 1 min). The bound cfDNA was washed three times with 0.5 mL QIAquick Buffer PE (Qiagen, Hilden, Germany) and 0.5 mL 95% ethanol and dried at 20000× *g* for 3 min. The cfDNA was eluted in 40 µL 10 mM Tris-Cl (pH 8.5).

The DNA concentration was measured using the Qubit^®^ 1X dsDNA HS Assay Kit and Qubit^®^ 4 fluorometer (Thermo Fisher Scientific, Waltham, MA, USA). The isolated plasma-derived and urinary cfDNA was subsequently stored at −80 °C until further analysis.

### 2.3. Whole-Genome Bisulfite Sequencing

To evaluate the performance of single- and double-stranded library preparation (ssLP and dsLP) for methylation analysis of cfDNA from urine and plasma, we used plasma cfDNA samples of two healthy volunteers and three liver transplant and four kidney transplant recipients. Further, urinary cfDNA samples from one healthy volunteer and one liver transplant recipient, extracted with both the Zymo Quick-DNA Urine kit and the Q Sepharose method, and from three kidney transplant and an additional two liver transplant recipients, extracted with the Q Sepharose method, were used (total: *n* = 18, Table S1).

For internal conversion quality control, 1 ng methylated pUC19 and 20 ng unmethylated lambda DNA (NEB, Ipswich, MA, USA) were sheared in 50 µL 10 mM Tris-Cl (pH 8.5) to 175 bp using the COVARIS LE220+ (Covaris Inc., Woburn, MA, USA). The sheared control DNA was diluted to 0.0037 ng/µL.

Single-stranded cfDNA libraries were prepared using the SRSLY NanoPlus kit (Claret Bioscience, Santa Cruz, CA, USA) with unique molecular identifiers (UMI) and unique dual indices (UDI). In short, 10 ng of cfDNA were spiked with 1 µL of diluted and sheared control DNA. The spiked cfDNA was denatured, and the single-stranded cfDNA fragments were ligated to methylated splint-adapters without end repair. The methylated adapters were provided by Claret Bioscience at the concentration for the SRSLY PicoPlus kit, which accommodates an input of up to 10 ng DNA. The adapter-ligated DNA was converted using the NEBNext Enzymatic Methyl-seq Conversion Module (NEB, Ipswich, MA, USA) by oxidizing 5-mC to 5-hmC and deaminated unmethylated cytosines. UMI and i7 index were added, followed by index PCR with eight cycles, simultaneously adding the i5 index. Clean-up of the amplified library was performed following the dual-sided SPRI bead DNA purification protocol. For the entire library preparation, Clarefy beads (Claret Bioscience, Santa Cruz, CA, USA) were used, and the small-fragment retention clean-up option was followed.

Double-stranded cfDNA libraries were prepared using the NEBNext Enzymatic Methyl-seq Kit (EM-seq^TM^; NEB, Ipswich, MA, USA). Here, approximately 35 ng of cfDNA was spiked with 1 µL of control DNA, followed by the steps described in the manual provided by the manufacturer. In detail, the cfDNA fragments were end repaired by filling up 5′ overhangs and digesting 3′ overhangs, followed by A-tailing. The end-repaired and A-tailed cfDNA was then ligated to methylated dT-tailed adapters, followed by enzymatic cytosine conversion. During the final PCR, unique dual indices were added, and the library was amplified with six cycles.

Library concentrations were determined with the Qubit™ dsDNA 1X HS Assay Kit (Thermo Fisher Scientific, Waltham, MA, USA), and the libraries were stored at −20 °C until pooling. 

The libraries were pooled, spiked with 5% of the PhiX Control v3 library (Illumina, San Diego, CA, USA) and clustered on an Illumina Novaseq 6000 SP flow cell, followed by paired-end (2 × 100 bp) sequencing on a Novaseq 6000 (Illumina, San Diego, CA, USA).

### 2.4. Bioinformatic Analysis

Demultiplexing was performed using the bcl2fastq Conversion Software v1.8.4 (Illumina, San Diego, CA, USA). Adapters were removed using *AdapterRemoval* v2.3.1, and reads were mapped to the hg19 genome and deduplicated (with the UMI sequence for the SRSLY samples) using *bismark* v0.23.0 [19]. The methylation status was extracted using *bismark* (--ignore 2 --no_overlap --report --no_header --gzip --bedGraph --zero_based --paired-end), with the additional flag “--ignore_r2 2” for the SRSLY data and the flag “--ignore_r2 5” for the EM-seq data. To directly compare the tissue proportions between the library preparation techniques, the BAM files for each EM-seq sample were down-sampled to reach the same average coverage as the BAM files from the SRSYL protocol using *samtools* v1.6. For TOO deconvolution, two algorithms and tissue atlases were used. 

For the individual CpG site-based deconvolution with 25 reference tissues, the atlas and deconvolution algorithm published by Erger et al. [13] was used (https://github.com/FlorianErger/cfNOMe, accessed on 20 June 2022), which uses the reference atlas published by Moss et al. [6] and the sequential least squares programming algorithm implemented in *scipy.optimize.minimize* [20] with a lower and upper bound of 0 and 1. With this algorithm, the sum of tissues does not necessarily add up to one, in contrast to the method from Moss et al.

For the fragment-level deconvolution, a novel reference atlas with 36 deep-sequenced FACS-sorted cell types and a non-negative least squares fragment-level deconvolution algorithm was used [21]. First, the read-level methylation information was extracted using *wgbstools* (https://github.com/nloyfer/wgbs_tools, accessed on 5 March 2023) from the deduplicated BAM file, using the default parameters, and deconvoluted using the UMX algorithm (https://github.com/nloyfer/UXM_deconv, accessed on 5 March 2023).

The Jagged Index-Unmethylated (JI-U) based on the mean CpG methylation by read position was calculated as proposed by Jiang et al. [22] and is defined as following, with M1 as the mean CpG methylation of the first 30 bp from the 5′ end of read 1 and M2 the mean CpG methylation of the first 30 bp at the 3′ of read 2:

(1) $$JI-U=\frac{M1-M2}{M1}\times 100\%$$

### 2.5. Statistical Analysis

All statistical analyses were performed using Python v.3.10. For multiple pairwise comparisons, the unpaired or paired *t*-test with Bonferroni correction was used. All figures were created using *matplotlib* v3.5.2 [23] and *seaborn* v0.11.2 [24].

## 3. Results

### 3.1. Within-Read Methylation Bias Differences between Library Preparation Techniques and cfDNA Source

**Figure A1:**
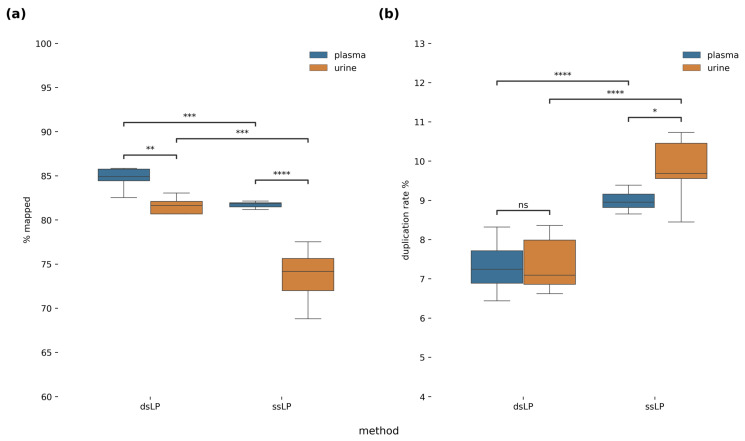
Percentage of (**a**) mapped reads and (**b**) duplication for urinary and plasma cfDNA with both library preparation methods (ssLP: *n* = 15; dsLP: *n* = 18). dsLP stands for double-stranded library preparation methods and ssLP for single-stranded library preparation methods. ns: *p* > 0.05; *: 0.01 < *p* ≤ 0.05; **: 0.001 < *p* ≤ 0.01; ***: 0.0001 < *p* ≤ 0.001; ****: *p* ≤ 0.0001.

All 18 extracted cfDNA samples were analyzed using the double-stranded method (dsLP), while 15 samples were also analyzed using the single-stranded method (ssLP). One urine and one plasma sample from two different kidney transplant recipients and one urine sample from a healthy volunteer were only analyzed with the dsLP method. An average of 39 mio. uniquely mapped and deduplicated clusters per sample were obtained for dsLP and 30 mio. for ssLP. The ssLP showed a significantly lower mapping rate compared to the dsLP method (78% vs. 83%, *p* < 0.01), while the mapping rate was lower for urine samples compared to plasma for both methods (ssLP: 74% vs. 82%, *p* < 0.001; dsLP: 81% vs. 85%, *p* < 0.01; Figure A1a). The duplication rate was higher for the ssLP method (9.4% vs. 7.3%, *p* < 0.0001), while it was higher for urine samples compared to plasma only for the ssLP method (9.8% vs. 9.0%, *p* < 0.05). The enzymatic conversion resulted in a similarly high mean conversion rate of 99.4% and 99.5% in combination with the single- and double-stranded library preparation kits, respectively. The mean methylation at CpG sites for the fully methylated pUC19 control DNA was high for both methods (ssLP: 96.6%; dsLP: 91.1%).

**Figure 2:**
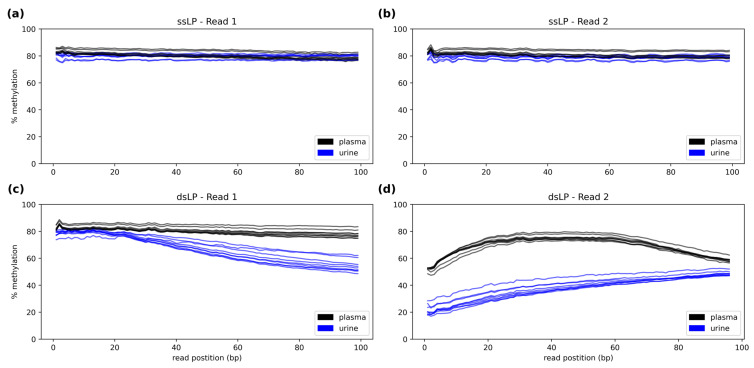
Within-read CpG methylation bias plot for the single-stranded (ssLP) and double-stranded (dsLP) library preparation method for both sequencing reads. Methylation bias plot for (**a**) read 1 and for (**b**) read 2 of ssLP samples (*n* = 15), and for (**c**) the read 1 and for (**d**) read 2 of the dsLP samples (*n* = 18).

The methylation proportion across each position in the read was analyzed separately for the forward and reverse read to assess within-read methylation bias. There was no bias observable for the ssLP across all read positions (Figure 2a,b). The dsLP, on the other hand, displayed a bias at the beginning and end of the second read for cfDNA extracted from plasma (Figure 2c,d). CfDNA from urine sequenced using the dsLP showed an even more pronounced bias for read 2, with a substantially lower proportion of CpG methylation at the beginning of the read, which continuously increased towards the end. While plasma cfDNA did not result in a visible bias for read 1, in urinary cfDNA, a decreasing CpG methylation was observed throughout almost the entire read for the dsLP, with an expected methylation rate only at the beginning of the read.

**Figure 3:**
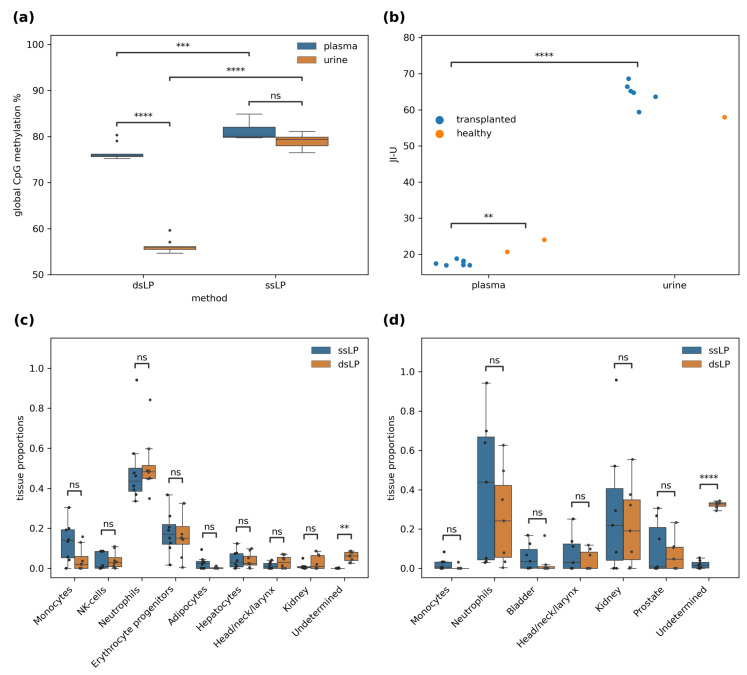
Differences between library preparation methods and body fluids in cfDNA methylation and tissue of origin results. (**a**) Global methylation difference between library preparation methods and cfDNA source (ssLP: *n* = 15; dsLP: *n* = 18). The black dots represent the outliers; (**b**) Jag index for cfDNA from urine and plasma. JI-U stands for Jagged Index-Unmethylated; Tissue of origin deconvolution results for plasma (*n* = 8) (**c**) and urinary (*n* = 7) (**d**) cfDNA with both library preparation methods. The black dots represent the individual sample values; dsLP: double-stranded library preparation method; ssLP: single-stranded library preparation method; (**a**,**b**) unpaired and (**c**,**d**) paired *t*-test; ns: *p* > 0.05; **: 0.001 < *p* ≤ 0.01; ***: 0.0001 < *p* ≤ 0.001; ****: *p* ≤ 0.0001.

The cfDNA methylation analysis from each body fluid revealed a similar overall CpG methylation rate for the ssLP approach (Figure 3a), with a median of 80.2% for plasma and 79.4% for urine. The dsLP, on the other hand, resulted in a much lower global methylation for urine samples (56.0%) compared to plasma (75.8%) (Figure 3a). Furthermore, the difference of the overall methylation of cfDNA between the library preparation methods was highly significant for both body fluids (Figure 3a). Methylation in the context of CHH (where H = A, T or G) was higher for the dsLP with 0.63% compared to the ssLP with 0.30% (*p* < 0.01).

Due to the end repair performed during dsLP with unmethylated cytosines, jagged ends of DNA fragments can be detected and quantified as the Jagged Index-Unmethylated (JI-U) proposed by Jiang et al. [22]. Urine showed an almost threefold increased value of 63.7 for the JI-U compared to 18.6 for plasma (Figure 3b). Moreover, the variance of the JI-U was small (0.5) for plasma cfDNA from transplant recipients as opposed to urinary cfDNA (9.6). In this study, plasma cfDNA of only two healthy volunteers was analyzed. However, the JI-U of both of these two samples was higher than in the seven cfDNA samples from the transplant patients (22.3 vs. 17.6).

### 3.2. Tissue of Origin Deconvolution Bias

**Figure A2:**
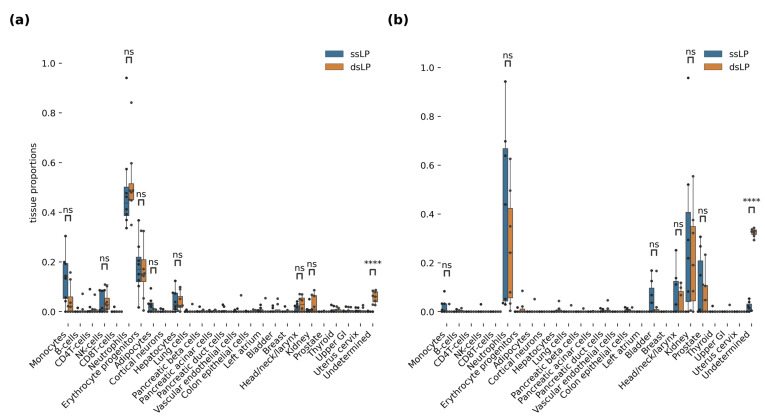
Deconvolution results for (**a**) plasma (*n* = 8) and (**b**) urinary (*n* = 7) cfDNA with both library preparation methods including all 25 tissues in the reference atlas. dsLP stands for double-stranded library preparation methods and ssLP for single-stranded library preparation methods. ns: *p* > 0.05; ****: *p* ≤ 0.0001.

Based on the methylation signature at 7890 informative loci and a reference atlas of 25 tissues, the TOO of the cfDNA was determined using a constraint linear least squares-based estimation. Limiting the sum of all tissues to 100% by an inequality constraint of one allowed a proportion of undetermined tissues. There was no significant difference between any tissue-specific proportion between the two library preparation techniques; however, there was a significant difference in the proportion of cfDNA with undetermined TOO in both body fluids (Figure 3c,d). This difference was much larger in urine (dsLP: 32.3% vs. ssLP: 1.9%, *p* ≤ 0.0001) than in plasma (dsLP: 5.9% vs. ssLP: 0.04%, *p* ≤ 0.0001) (Figure 3d). Due to the potential lack of sensitivity for rare cell types, we only analyzed the top eight tissues based on their mean proportion in plasma and the top six in urine. The median proportion of cfDNA from one cell type, erythrocytes progenitors, was high in plasma (dsLP: 0.15; ssLP: 0.18), but very low or undetectable in urine, where only two samples where above zero with the dsLP and one with the ssLP (Figure A2).

**Figure A3:**
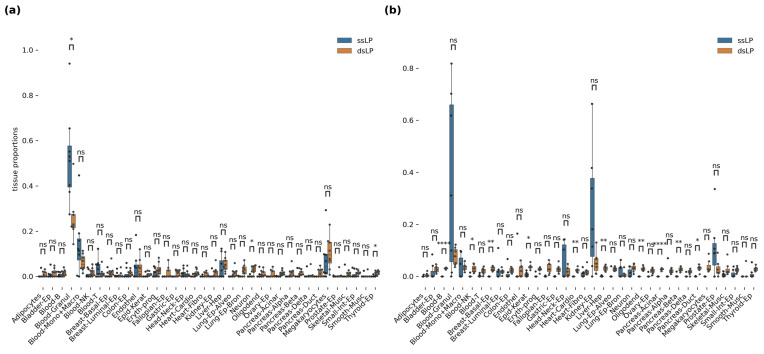
Deconvolution results of the fragment-level UXM algorithm and 36 reference tissues/cell types for urinary and plasma cfDNA with both library preparation methods. Displayed are the cell type proportions for the cfDNA from (**a**) plasma (*n* = 8) and (**b**) urine (*n* = 7). dsLP stands for double-stranded library preparation methods and ssLP for single-stranded library preparation methods. paired *t*-test: ns: *p* > 0.05; *: 0.01 < *p* ≤ 0.05; **: 0.001 < *p* ≤ 0.01; ****: *p* ≤ 0.0001.

Fragment-level instead of individual CpG site-based deconvolution represents a different approach to infer the proportions of different TOO of cfDNA. To evaluate the effects of the different library preparation methods on the fragment-level deconvolution results, a novel reference atlas comprised of 36 different cell types and a fragment-level deconvolution algorithm [21] was used. When comparing the two library preparation methods, about 8% of the cell type proportions (*n* = 3 cell types) were significantly higher in plasma when sequenced with the dsLP and deconvoluted using the fragment-level algorithm, while 28% of the cell type proportions (*n* = 10 cell types) were higher in urine (Figure A3). It is worth highlighting that almost exclusively, the affected cell types had a proportion of mostly zero for the site-based approach and for the fragment-level approach in data from the ssLP method.

## 4. Discussion

To our knowledge, this is the first study investigating the effects of different library preparation approaches on the TOO deconvolution of cfDNA. The findings in this study outline potential biases associated with single-strand and double-strand library preparation methods specifically for methylome analysis of cfDNA from urine and plasma, the two most frequently investigated sources of circulating nucleic acids.

The estimation of the genome-wide average CpG context methylation resulted in a lower methylation for urinary compared to plasma cfDNA for the double-stranded library preparation, with 75% in plasma and 56% in urine. These findings are in agreement with the only known study applying a double-stranded library preparation method in conjunction with enzymatic conversion to cfDNA from urine and plasma by Erger et al. [13], which found an average CpG methylation of plasma-derived cfDNA of about 75% and just above 60% for urinary cfDNA from healthy individuals (*n* = 3). Such a difference in average CpG methylation of cfDNA between body fluids is not expected to have a biological origin. However, the observation of a more pronounced methylation bias at the ends of sequencing reads from urinary cfDNA is in accordance with the end repair procedure creating blunt ends from 5′ overhangs in the double-stranded protocol. The incorporation of unmethylated cytosines at the 3′ ends of the reverse strands leads to a lower average methylation, explaining the observed bias in overall methylation. In the single-stranded library preparation approach, on the other hand, this bias is avoided, as end repair is not needed for adapter ligation. The methylation bias data from the dsLP suggests that the length of the jagged ends in urine can range from fully double-stranded to almost fully single-stranded. For the observed lack of methylation bias at the beginning of dsLP read 1, it is currently not known whether this is due to a biological or technical reason, such as the loss of jagged end fragments with a shorter double-stranded section during end repair.

Despite the overall methylation bias for the double-stranded methods, the TOO proportions did not differ significantly for both urine- and plasma-derived cfDNA, suggesting that cfDNA originating from different tissues is equally affected by this bias. The proportion of undetermined tissues, however, was greatly increased in both body fluids, with a stronger increase in urine for the dsLP, which could suggest a higher proportion of unknown cell types. However, considering the methylation bias, and as the dsLP is unlikely to specifically capture an increased proportion of cfDNA from unknown cell types, this increase is likely almost exclusively attributable to non-biological methylation patterns at the individual CpG sites used for deconvolution that are introduced through the end repair. Interestingly, the effects of the bias on the deconvolution results appeared more significant when a fragment-level-based deconvolution algorithm was used. With this approach, instead of increasing the undetermined TOO proportion, the dsLP resulted in increased fractions of rare cell types, which likely originated from incorrect TOO assignment.

The severity of the methylation bias introduced by end repair is heavily dependent on the length of single-stranded overhangs, as well as on the frequency of cfDNA with overhangs among the bulk cfDNA fragments. Leveraging this bias in the dsLP, we showed that urinary cfDNA displays increased jaggedness compared to plasma-derived cfDNA. These findings are in agreement with previous findings from Zhou and colleagues [25]. While both studies observed increased jaggedness in urinary cfDNA compared to plasma cfDNA, the median JI-U of 67.5 in this study was about twice as high compared to the value of 32 reported in Zhou et al. Different urinary cfDNA extraction methods were used in the two studies and might explain in part the difference in JI-U, as with increasing length of overhangs, cfDNA fragments behave more closely to ssDNA, which shows different properties in regard to silica surface binding [26]. As a consequence, either proportionally more jagged fragments and/or fragments with longer overhangs may have been extracted by the method used here. 

Interestingly, for the jagged end analysis in plasma cfDNA, a significant difference in the JI-U between transplant recipients and healthy volunteers was observed. It is important to note that cfDNA from only two healthy volunteers was sequenced here, and thus, statistical power is limited. However, we believe that based on the results in this pilot study, combined with its potential as a biomarker in other diseases [25,27], further investigation of cfDNA jaggedness in transplant recipients might be worth pursuing, as it has been largely unexplored so far. Moreover, the mechanism underlying such a difference between healthy individuals and transplant recipients is currently unknown.

Besides the small sample size for the jagged end analysis of healthy volunteers and transplant recipients, another potential limitation of this study is that the samples were sequenced at a low coverage, which cannot be used for a reliable library complexity estimation. Further, it also limits the detection of differences between the library preparation methods regarding sensitivity and biases for the detection and quantification of low-abundance cell types among the cfDNA, which would require deeper sequencing. Lastly, because of the limited sample size, small differences of the TOO proportions between the two library preparation methods cannot be excluded, but might still be biologically relevant, depending on the context of the TOO investigation.

In conclusion, this study outlines potential biases and associated noise introduced by end repair in the double-stranded library preparation for methylation analysis of cfDNA, especially if derived from urine, which are avoided using a single-stranded approach. If the metrics of interest are TOO proportions and preservation of end motifs, a single-strand library preparation in conjunction with a site- or fragment-level deconvolution algorithm is likely the recommended combination. However, if jagged end analysis is also desired besides tissue proportions, a double-stranded method should be used with site-based deconvolution. However, given the substantial jaggedness in urinary cfDNA, the application of this approach is likely limited to plasma-derived cfDNA in order to avoid an extensive methylation bias. As such, our findings will inform the design of future studies of cfDNA TOO using whole-genome methylation sequencing.

## Data Availability

The raw FASTQ files have been deposited at the European Genome-phenome Archive (EGA) under the study accession number: EGAS00001007314. Access to the data is possible through application to the responsible Data Access Committee (DAG).

## Supplementary Materials

The following supporting information can be downloaded at: https://www.mdpi.com/article/10.3390/diagnostics13152505/s1, Table S1: Overview of extracted and sequenced samples.
